# MMP-2 and TIMP-1 predict healing of WTC-lung injury in New York City firefighters

**DOI:** 10.1186/1465-9921-15-5

**Published:** 2014-01-21

**Authors:** Anna Nolan, Sophia Kwon, Soo Jung Cho, Bushra Naveed, Ashley L Comfort, David J Prezant, William N Rom, Michael D Weiden

**Affiliations:** 1 Division of Pulmonary, Critical Care and Sleep, New York University, School of Medicine, 462 First Avenue, NB7N24, New York NY, USA; 2 Department of Environmental Medicine, New York University, School of Medicine, 57 Old Forge Road, Tuxedo Park NY, USA; 3 Fire Department of New York, Bureau of Health Services and Office of Medical Affairs, Brooklyn NY, USA; 4 Department of Medicine, Pulmonary Medicine Division, Montefiore Medical Center and Albert Einstein College of Medicine, Bronx NY, USA; 5 Department of Medicine and Environmental Medicine, 550 1st Avenue, New York, NY 10016, USA

**Keywords:** Biomarkers, Lung disease, Occupational exposure

## Abstract

**Rationale:**

After 9/11/2001, most FDNY workers had persistent lung function decline but some exposed workers recovered. We hypothesized that the protease/anti-protease balance in serum soon after exposure predicts subsequent recovery.

**Methods:**

We performed a nested case–control study measuring biomarkers in serum drawn before 3/2002 and subsequent forced expiratory volume at one second (FEV_1_) on repeat spirometry before 3/2008. Serum was assayed for matrix metalloproteinases (MMP-1,2,3,7,8,9,12 and 13) and tissue inhibitors of metalloproteinases (TIMP-1,2,3,4). The representative sub-cohort defined analyte distribution and a concentration above 75^th^ percentile defined elevated biomarker expression. An FEV_1_ one standard deviation above the mean defined resistance to airway injury. Logistic regression was adjusted for pre-9/11 FEV_1_, BMI, age and exposure intensity modeled the association between elevated biomarker expression and above average FEV_1_.

**Results:**

FEV_1_ in cases and controls declined 10% of after 9/11/2001. Cases subsequently returned to 99% of their pre-exposure FEV_1_ while decline persisted in controls. Elevated TIMP-1 and MMP-2 increased the odds of resistance by 5.4 and 4.2 fold while elevated MMP-1 decreased it by 0.27 fold.

**Conclusions:**

Resistant cases displayed healing, returning to 99% of pre-exposure values. High TIMP-1 and MMP-2 predict healing. MMP/TIMP balance reflects independent pathways to airway injury and repair after WTC exposure.

## Introduction

The collapse of the World Trade Center (WTC) on 9/11/2001 produced a massive exposure to dust and products of combustion [1-3]. The Fire Department of New York (FDNY) bureau of health services rapidly responded to this atrocity, initiating a medical monitoring program in October of 2001. Over 13,000 exposed rescue workers have been longitudinally followed by the FDNY-WTC-Medical Monitoring and Treatment Program. Approximately 7,000 exposed workers had serum samples drawn, stored and FEV_1_ and FVC measured within six months of 9/11/2001. A vast majority of those exposed had an acute decline in lung function in the first six months followed by stabilization. There was no recovery in lung function for the group as a whole, but a minority of those exposed recovered lung function over the following six and a half years.

We used serum obtained soon after the exposure to measure serum biomarkers of lung injury during the process of disease evolution. We reported that inflammatory cytokines, lipids and other measure of metabolic syndrome as well as biomarkers of cardiovascular risk predict abnormal lung function years later [4-6].

The balance of increased protease activity and reduced anti-protease activity are components of many diseases including cigarette-induced chronic lung disease and other causes of accelerated lung function decline [7-10]. Genetic association studies with matrix metalloproteinases (MMPs) demonstrate a strong association with the development of lung disease [7]. MMP-1 is induced in smokers with COPD and its overexpression in mice causes emphysema [11,12]. The destructive effects of MMPs are inhibited by tissue inhibitors of matrix metalloproteinases (TIMPs). Since most clinical investigation focuses on disease, there is little data on the role of MMPs and TIMPs in the resistance to the damaging effect of dust exposure [13-16]. One carefully done pathologic study demonstrated increased MMP-2 and TIMP-1 mRNA expression in surgically removed lung and predicted improved FEV_1_ in COPD patients [17]. Serum MMP and TIMP expression reflects the severity of COPD supporting an investigation of a link between serum MMP/TIMP balance and lung function in the WTC exposed cohort [18].

This study investigates serum expression of MMPs and TIMPs soon after damaging particulate matter exposure and tests if protease/anti-protease balance is associated with a subgroup that demonstrates an above average healing potential after WTC-LI. We report that elevated TIMP-1 and MMP-2 predicts recovery of lung function while elevated MMP-1 reduces the odds of recovery years after WTC exposure.

## Methods

### Study participants and design

The Institutional Review Boards (IRB) of NYU and Montefiore Medical Center approved this study and patients signed consent at the time of serum draw within 6 months of 9/11/2001 (Montefiore Medical Center IRB; #07-09-320 and New York University IRB; #11-00439). The parent cohort for this investigation consists of 1,720 exposed workers who required subspecialty pulmonary examination (SPE) prior to 3/10/2008.

A nested case–control study tested the association of serum biomarkers and FEV_1_ at SPE. The baseline cohort N = 801 was assembled to exclude patients with abnormal pre-9/11 FEV_1_ and tobacco use to eliminate these confounders of post exposure lung function [19]. Resistant cases (N = 100/801) had a FEV_1_% one standard deviation above the mean (>107%) at SPE. Controls (N = 171/801) were randomly selected after stratification of the baseline cohort for BMI and FEV_1_. Serum was available for N = 137/171 of the cohort controls, N = 77/100 of the resistant cases. For this case–control study, the control subjects are all individuals in the random sample cohort control who did not meet criteria to be resistant cases. Analyte distribution in the cohort controls identified the 75^th^ percentile cut points used to define elevated biomarker expression.

### Serum biomarker assays

Processing of blood has been previously described [6,20]. Biomarkers were assayed with a TIMP panel (R&D Systems, Minneapolis, MN), and MMP panel (Procarta/Affymetrix) using a Luminex 200-IS (Luminex Corporation, Austin, TX). Each plate contained 1:2 ratio of resistant cases to controls to account for batch effect.

#### Chest computed tomography (CT)

We have reported on lung function and computed tomography (CT) findings of this group. High-resolution chest CT scans were obtained with 7-mm thick mages reconstructed at 6-mm intervals. Images were read for airway and parenchymal abnormalities by dedicated, board certified radiologists who had no knowledge of the subjects’ exposure status or clinical findings. Bronchial wall thickening and air-trapping were interpreted qualitatively and analyzed as a yes/no variable.

### Statistical analysis

Database management and statistics used SPSS 20 (IBM, Armonk, NY). Odd ratios were modeled with multivariate binary logistic regression with case status as the outcome. Analyte cutoff values were the 75^th^ percentile cohort control expression. The Hosmer-Lemeshow goodness-of-fit test was used to assess calibration of the final model. The model discrimination was evaluated using the receiver operating characteristic area under the curve (AUC).

## Results

### Participants

**Figure 1:**
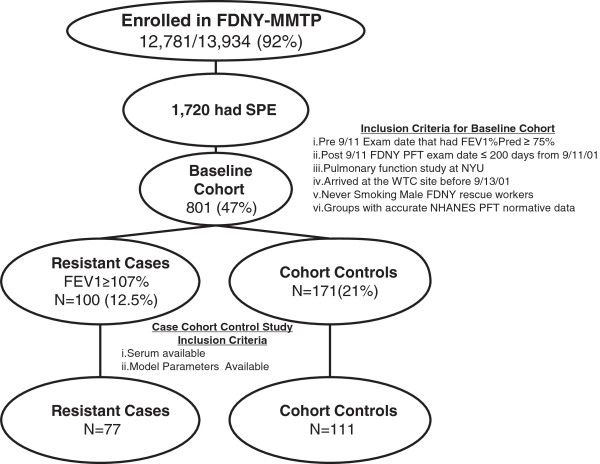
**Study design.** Derivation of cases n = 77 and controls n = 111 from the FDNY-MMTP.

Derivation of cases and controls is depicted in Figure 1. N = 111/171 cohort controls with serum available for study were compared to N = 77/100 resistant cases. Controls were not significantly different from the baseline cohort in arrival time, years of service, age, time to all pulmonary examinations, or BMI, Table 1. Controls had higher BMI than resistant cases. Time to medical monitoring entry (MME), when spirometry was performed and serum drawn, was 2 months in cases and controls. Time to subspecialty pulmonary evaluation SPE, when spirometry that defined case status was performed, was 32 months in cases and controls.

**Table 1:** Demographics

	**Date/event**	**Baseline cohort**	**Sub-cohort controls**	**Resistant cases**	**p**
WTC Exposure, n(%)	High	197 (25)	21 (19)	14 (18)	1.000
	Intermediate	604 (75)	90 (81)	63 (82)	
9/11 to PFT, months	MME	2.7 (2.0-3.8)	2.5 (2.0-3.2)	2.6 (2.2-3.2)	0.219
	SPE	33.8 (24.8-57.0)	32.7 (24.6-57.4)	32.5 (28.3-52.2)	0.755
BMI, kg/m2	MME	28.0 (26–30)	28.1 (26–31)	27.3 (26–29)	0.018
	SPE	28.9 (27–31)	29.1 (27–31)	27.6 (26–30)	0.010
Years of service	9/11/01	13 (7–19)	14 (7–18)	14 (10–19)	0.249
Age	9/11/01	40 (36–45)	41 (36–44)	42 (38–46)	0.159

*WTC* World trade center, *PFT* Pulmonary function test, *BMI* Body mass index, *MME* Medical monitoring entry, *SPE* Subspecialty pulmonary exam.

Expressed as median (interquartile range.

### Longitudinal lung function in resistant cases and controls

**Figure 2:**
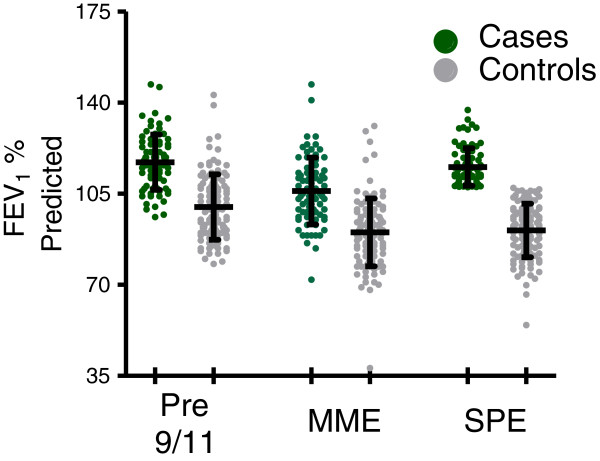
**FEV**_**1**_**% predicted of cases and controls over time.** Mean (SD) expressed for cases n = 77 (green) and controls n = 111 (grey) at Pre-9/11, MME and SPE.

Cases and controls underwent three longitudinal measures of lung function. The first spirometry documented pre-exposure lung function; the second was immediately after exposure at MME and the third was later at SPE, Table 2 and Figure 2. Resistant cases had higher median pre-9/11 FEV_1_ than controls (117% vs 98% p < 0.001).

**Table 2:** Longitudinal lung function assessment of cohort

**Time**	**Variable**	**Controls**	**Resistant**	**p**
Pre-9/11	FEV1%	98 (90–108)	117(108–124)	<0.0001
	FEV1/FVC	84 (81–87)	87 (84–89)	<0.0001
MME	FEV1%	91 (81–96)	105(97–113)	<0.0001
	FEV1/FVC	84 (79–87)	85 (83–89)	<0.0001
SPE	FEV1%	93 (83–98)	113 (109–119)	<0.0001
	FEV1/FVC	77 (73–79)	81 (79–84)	<0.0001
	BD response	5 (2–10)	5(2–9)	0.776
	BD response > 12%, N (%)	13/46 (28)	5/23 (22)	0.772
	MCT slope	0.64 (0.31-0.15)	0.04(0.02-0.07)	0.010
	PC20 < 10 mg/mL MCT, N (%)	17/86 (20)	6/66 (9)	0.108
	TLC	103 (95–109)	106 (103–116)	0.016
	RV,% Pred	125 (113–141)	111 (103–123)	0.015
	DLCO	106 (99–114)	116 (108–131)	0.001
	VA	93 (85–98)	100 (95–105)	<0.0001
	DLCO/VA	120 (107–130)	110 (107–126)	0.358

*FEV1* Forced expiratory volume in one second, *FVC* Forced vital capacity, *MCT* Methacholine, *BD* Bronchodilator, *PC20* concentration of methacholine causing a 20% fall in FEV1, *DLCO* Diffusion capacity of the lung for carbon monoxide, *VA* Alveolar volume.

FEV_1_ declined in cases and controls soon after exposure. To test if group data represented individuals’ response the ratio of FEV_1_ pre-9/11 to FEV_1_ at MME was calculated for each case and controls. The mean (SD) of MME/Pre-911 ratio was 0.91(0.10) for resistant cases and 0.91 (0.12) for controls (p = 0.95 for cases vs control). Cases recovered most of the lost FEV_1_ (117% to 113%). Controls had less improvement than cases in their median FEV_1_ (98% to 93%). To test if group data represented individuals’ response the ratio of FEV_1_ pre-9/11 to FEV_1_ at SPE was calculated for each case and controls. The SPE/Pre-911 ratio was 0.99+/-0.09 for resistant cases and 0.91+/-0.12 for controls (p < 0.001 for cases vs control). Raw FEV_1_% predicted has also been shown in Figure 2.

Both groups had a decline in FEV_1_/FVC ratio SPE/MME, with resistant cases having the less than controls (0.85 to 0.81 p < 0.001). Both cases and controls had a high proportion of individuals with bronchodilator response (22% vs 28% NS). Cases had less methacholine reactivity when measured as a continuous slope (0.64 vs 0.04 p < 0.01); 9% of cases reactive to a 10 mg/ml dose while 20% of controls were reactive (p = 0.11). Cases had less air trapping than controls (111% vs 121% p < 0.02) when residual volume was used to measure air trapping Table 3. At SPE, resistant cases had the higher TLC, DLCO and alveolar volume than controls (p < 0.001 for all comparisons), Table 2.

**Table 3:** Chest CT abnormalities in cases and controls

**CT Finding**	**Controls**	**Resistant cases**	**p†**
Bronchial wall thickening	23/65 (35)	5/36 (14)	0.022
Air trapping	30/65 (46)	10/36 (28)	0.090
Nodules	25/65 (38)	13/36 (36)	0.834

^†^Significance calculated by Chi-Squared.

### Chest CT of resistant cases and controls

Chest imaging was used to investigate if resistant cases and controls had structural differences. In those with chest CT images available, 14% of the resistant cases had bronchial wall thickening whereas 35% of the controls had this evidence of airway inflammation (p < 0.03), Table 3. There was no significant difference between cases and controls in air trapping defined by mosaic attenuation (28% vs 46% p = 0.09). Both cases and controls have a high proportion of pulmonary nodules (38% vs 36% p = 0.8).

### Biomarker models

Analyte levels were compared between controls and resistant cases, Table 4. We used logistic regression with analyte expression above a pre-defined 75^th^ percentile cut points to test if protease or anti-proteases expression predicted resistance to WTC-LI [21]. Models were adjusted for BMI, age, exposure group and pre-9/11 FEV_1_. Reduced models examined the ability of a single analyte to predict each case definition. Analytes with significant odds ratios in single biomarker models were used to develop the final multi-analyte model.

**Table 4:** Serum biomarkers

	**Analyte pg/mL**	**75th percentile cutpiont**	**Controls**		**Resistant**		**OR (95% CI)**
			**Median (IQR)**	**Proportion**	**Median (IQR)**	**Proportion**	
Proteases	MMP-1	1239	689 (264–1210)	27/111	549 (233–1013)	14/77	0.691 (0.335-1.425)
	MMP-2	4949	2933 (1570.01-4608.25)	22/111	3340 (2108.19-5682.13)	27/77	2.185 (1.128-4.231)
	MMP-3	13345	7494 (2940–13358)	28/111	6906 (3457–11509)	17/77	0.840 (0.422-1.671)
	MMP-7	355	217 (100–333)	25/111	237 (95–375)	22/77	1.376 (0.707-2.-77)
	MMP-8	157	10 (10–156)	27/111	10 (10–116)	16/77	0.816 (0.405-1.645)
	MMP-9	62086	22559 (10596–40806)	24/111	23003 (12043–64398)	21/77	1.359 (0.692-2.670)
	MMP-12	306	66 (18–306)	28/111	55 (27–277)	19/77	0.971 (0.496-1.902)
	MMP-13	138	75 (10–133)	26/111	71 (10–140)	20/77	1.147 (0.585-2.248)
Anti-proteases	TIMP-1	155003	125491 (100396–151151)	23/111	126292 (100483–164661)	29/77	2.312 (1.206-4.430)
	TIMP-2	115905	102470 (89901–113738)	23/111	103199 (88959–119820)	22/77	1.530 (0.780-3.005)
	TIMP-3	31922	7750 (7750–31922)	29/111	7750 (7750–33680)	21/77	1.060 (0.550-2.044)
	TIMP-4	1585	1242 (9288–1562)	26/111	1342 (970-(1717)	27/77	1.765 (0.929-3.354)

*MMP* matrix metalloproteinase, *TIMP* Tissue inhibitor of matrix metalloproteinase, *OR* Odds RATIO (unadjusted), *IQR* Interquartile range.

**Figure 3:**
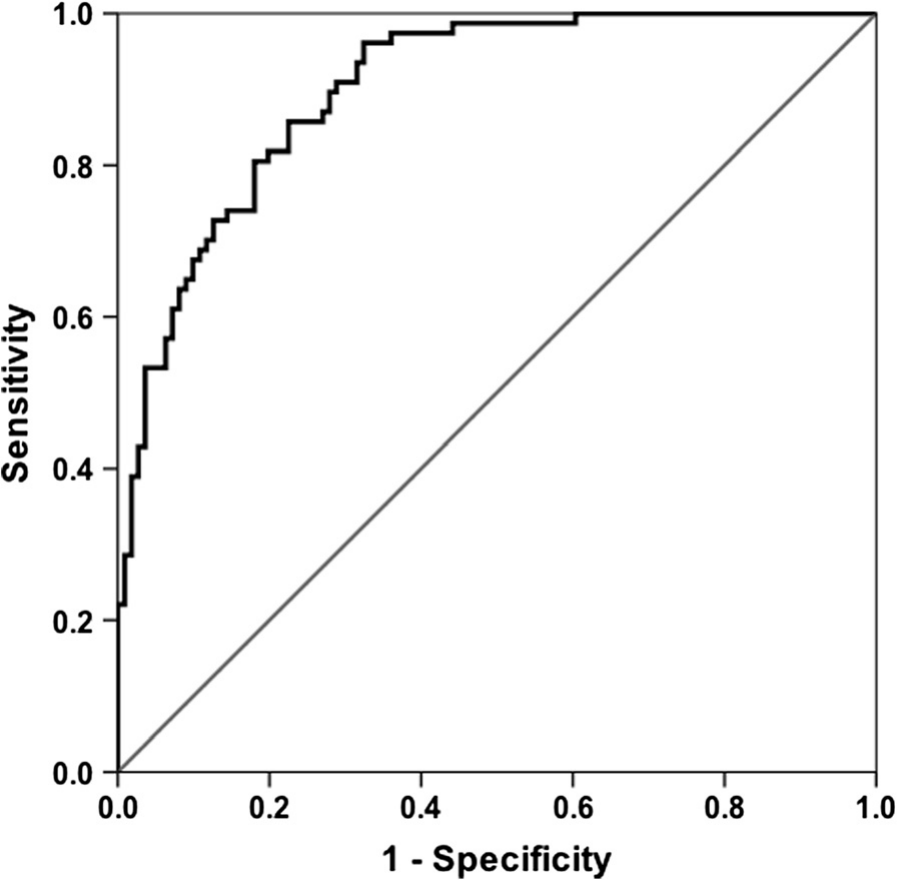
**Receiver operator characteristics (ROC) of final multi-analyte model.** Area Under the Curve (AUC) = 0.901: 95% CI (0.860-0.943).

Using 75^th^ percentile of cohort expression, we calculated OR with FEV1 > 107% at SPE as the outcome. Elevated MMP-1 reduces the odds of FEV1 > 107% by 31%, elevated MMP-2 increases the odds of FEV1 > 107% by 218% and elevated TIMP-1 increases the odds by 231%. After adjusting for BMI, age, exposure group and pre-9/11 FEV_1_ elevated MMP-1 reduces the odds of resistance to WTC-LI by 68% while individuals with high MMP-2 and TIMP-1 were 300% and 350% more likely to resist WTC-LI, Table 4. Combining MMP-1, MMP-2 and TIMP-1 in a multi-analyte model improved the OR for each of these biomarkers, Table 5. Elevated MMP-1 was a risk factor reducing the odds of resistance to lung injury to 0.27 (95% CI 0.09-0.82 p = 0.02). Elevated MMP-2 and TIMP-1 were protective factors improving odds of resistance by 4.2 fold (95% CI 1.6-10.8 p = 0.003) and 5.4 fold (95% CI 1.9-14.9 p = 0.001), Table 5. The area under the receiver operator curve for the multi-analyte model was 0.90 (95% CI; 0.86-0.94). (Figure 3) The sensitivity and specificity of the model was 74% and 86% respectively.

**Table 5:** Model of resistance to WTC-lung injury

	**Serum biomarker**	**OR (95% CI)**
	**75th percentile cutpoint**	**Adjusted§**
Single analyte	MMP-1 ≥ 1239	0.33 (0.11-0.93)
	MMP-2 ≥ 4949	3.00 (1.25-7.18)
	TIMP-1 ≥ 155003	3.52 (1.41-8.81)
Multi-analyte	MMP-1 ≥ 1239	0.27 (0.09-0.82)
	MMP-2 ≥ 4949	4.16 (1.61-10.76)
	TIMP-1 ≥ 155003	5.38 (1.94-14.94)

§Models Adjusted for: BMI at SPE, exposure Group, Age at 9/11 and Pre-9/11 FEV_1_% Predicted; -2 Log Likelihood 144.081; Hosmer and Lemeshow (Goodness of Fit) Sig 0.743.

## Discussion

We report that elevated MMP-1, MMP-2 and TIMP-1 in serum shortly after WTC exposure predicts subsequent return of FEV_1_ to pre-exposure values. All subjects in this nested case control investigation had significant WTC dust exposure, arriving at the collapse site within 2 days of 9/11/2001. The FDNY measured serial FEV_1_ pre and post 9/11. We focused our current study on a subgroup of highly exposed individuals who did not suffer persistent FEV_1_ decline. This resistant subgroup had greater than average reduction in FEV_1_ immediately after exposure but returned to pre-exposure FEV_1_ over the next 6.5 years. Because serum was drawn well before the pulmonary function test that demonstrated recovery, the biomarker information reflected the evolving response to injury. MMP-2 and TIMP-1 expression above the 75^th^ percentile are protective biomarkers, significantly increasing the odds of resistance between 4.2 and 5.4 fold. Alternately, elevated MMP-1 is a risk factor, reducing the odds of resistance by 73%. The biomarker model using serum MMP-1, MMP-2 and TIMP-1 concentration predicted resistance with a sensitivity of 74%, a specificity of 86% and a receiver operator characteristic of 0.90.

As expected in this highly exposed group, both cases and controls suffered an acute reduction in FEV_1_ as a result of WTC exposure. Resistant cases differed from controls because they returned to 99% of their pre-exposure FEV_1_. Over the 6.5 years post 9/11 FEV_1_ returned to only 91% of their pre-exposure FEV_1_. The return of FEV_1_ to pre-exposure levels provides evidence that resistant cases have above average capacity to heal after an acute injury.

Elevated TIMP-1 and MMP-2 expression increases the odds of being resistant 4.1 and 5.3 fold respectively. These results remain significant after adjusting for multiple comparisons. Although TIMP and MMP over expression have been observed in COPD and are probably affected by chronic injury secondary to cigarette smoke or other damaging processes, interpreting the cause and effect relationship between lung function and MMP/TIMP balance in humans has been challenging [22-24]. A carefully done study of 63 patients who had surgery for lung cancer or lung transplantation demonstrated that increasing TIMP-1 and MMP-2 mRNA in the small airways and/or the parenchyma surrounding the small airway was positively associated with FEV_1_[17]. Our findings on particulate matter induced lung injury are consistent with these findings in smoking related COPD patients. The mechanisms involved in the protective effects of TIMP-1 and MMP-2 in response to particulate matter induced lung injury require further study.

Since resistant cases were defined by a FEV_1_ of > 107%, starting close to this threshold should increase the likelihood of crossing this defining value. After adjusting the logistic model for pre-9/11 FEV1, the association of MMP-1 MMP-2 and TIMP-1 with better than average post exposure FEV_1_ markedly improved as quantified by their ORs. This suggests that while pre-existing differences in lung function is a confounder including them in the regression allowed the biomarker-lung function association to remain robust. We briefly examined if excluding individuals who failed their MCT by the 3^rd^ level would alter our results. We found that the ORs of MMP-1, MMP-2 and TIMP-1 remained significant and of the same magnitude (data not shown). Furthermore, we repeated the analysis in a population with their Pre-9/11 FEV_1_% predicted constrained; cases n = 58 had an FEV_1_ of 96–146 and controls n = 58 had an FEV_1_ of 96–143. Using this constrained population OR (95% CI) were as follows: MMP-1 0.318 (0.098-1.039), MMP-2 4.993 (1.664-14.983) and TIMP-1 8.061 (2.423-26.825). Importantly, patients with resistance to WTC-LI had at subspecialty pulmonary evaluation increased TLC measured on plethysmography, increased alveolar volume measured by methane dilution and increased diffusion of carbon monoxide than controls. In animal models, MMP-2 is required for normal lung development and failure to produce MMP-2 leads to emphysema and collagen deposition around the bronchioles [25-27]. Our data is consistent with the hypothesis that MMP-2 and TIMP-1 are biomarkers of an individual’s intrinsic capacity to heal after irritant induced lung injury.

The resistant cases also had significantly less bronchial wall thickening on chest CT. Accumulation of inflammatory cells in the bronchovascular bundle likely accounts for this radiographic finding [28]. Our Chest CT findings suggest that the resistant group accumulates fewer inflammatory cells around the bronchovascular bundle after dust exposure. Interestingly, in rodent models of lung injury, MMP-2 expression reduces bronchovascular inflammatory cells and enhances repair [29,30]. Since the chest CT was performed years after the insult, the mechanism that produced the bronchial wall thickening persists for years after the original exposure that precipitated the inflammation.

Conversely, elevated MMP-1 reduced the odds of being resistant to WTC-LI to 0.27. This association, however, becomes insignificant when adjusted for multiple comparisons. We have maintained MMP-1 in the model because of the overwhelming evidence that this protease is an important is disease pathogenesis. Over expression of MMP-1 leads to emphysema in animal models [31]. In humans, MMP-1 is expressed in type II pneumocytes of patients with COPD but not controls [11,12]. The 73% reduction in the odds of having above average FEV_1_ is consistent with the damaging effects of this protease on lung integrity.

Since we excluded ever smokers and individuals with pre-9/11 lung disease, our results are not confounded by these common causes of FEV_1_ decline that are unrelated to inhalation of WTC dust. Another advantage of this study is the SPE PFT was performed during the initial pulmonary evaluation prior to treatment initiation. Therefore our results are not confounded by treatment effect. In spite of our objective case definition, using FEV_1_ as a single measure did produce misclassification of disease. The resistant group with FEV_1_ > 107% at SPE did have a individuals with evidence of airway injury. Up to 22% of the resistant cases had airway reactivity on PFT or radiographic evidence of bronchial wall thickening. This misclassification of disease should bias toward the null. In spite of this bias, we observed highly significant associations between biomarkers in serum drawn soon after exposure and above average lung function years later.

This nested case control study has several limitations. The cohort was highly unusual, suffering an acute overwhelming exposure to PM that overwhelmed normal protective mechanisms. The results therefore have limited generalizability. The findings require replication in independent particulate matter exposed cohorts. Even though the serum biomarkers were expressed years before the FEV_1_ that defined resistance to lung injury, the results are correlations and do not imply causation. It is possible that exposure lead to an alteration in these biomarkers due to different mechanisms controlling lung injury. Alternately, pre-existing attenuation of these biomarkers may lead to differential healing. Further, investigation in model systems and longitudinally followed cohorts is required to better understand the role, if any, of MMP-1 and TIMP-1 in healing after PM induced lung injury. Finally, this study had no unexposed control group because the few unexposed workers were markedly different from the exposed group with poor health that prevented them from working at the WTC collapse site. This restricts our ability to assess the impact of WTC exposure to the observed biomarker disease relationship.

This report documents the serum biomarkers that predict better than average FEV_1_ after massive dust exposure. This group had evidence of healing with return to pre-9/11 FEV_1_ after a significant drop immediately post exposure. The processes initiated by WTC exposure impacted multiple distinct injury and repair pathways. One interpretation of the findings is that biomarkers of resistance reflect biological processes leading to healing after particulate matter induced injury.

## Competing interests

The authors report no financial or competing interests.

## Authors’ contributions

AN, SK and MDW participated in study conception and design. AN, SK and MDW were the primary investigators. AN, SK, ALC, BN and MDW were responsible for data collection. AN, SJC and SK were responsible for data validation. AN, ALC and SK participated in data analysis. AN, SK undertook the statistical analysis. All authors participated in data interpretation, writing and revision of the report and approval of the final version. All authors read and approved the final manuscript.

## References

[B1] Landrigan PJ Health consequences of the 11 September 2001 attacks Environ Health Perspect 2001 109 11 A514 A515 Epub 2001/11/20 10.1289/ehp.109-a514 11713006 PMC1240482

[B2] Claudio L Environmental aftermath Environ Health Perspect 2001 109 11 A528 A536 Epub 2001/11/20 10.1289/ehp.109-a528 11713010 PMC1240484

[B3] Aldrich TK Gustave J Hall CB Cohen HW Webber MP Zeig-Owens R Lung function in rescue workers at the World Trade Center after 7 years N Engl J Med 2010 362 14 1263 72 Epub 2010/04/09 10.1056/NEJMoa0910087 20375403 PMC4940972

[B4] Weiden MD Naveed B Kwon S Segal LN Cho SJ Tsukiji J Comparison of WTC dust size on macrophage inflammatory cytokine release in vivo and in vitro PLoS One 2012 7 7 e40016 Epub 2012/07/21 10.1371/journal.pone.0040016 22815721 PMC3399845

[B5] Naveed B Weiden MD Kwon S Gracely EJ Comfort AL Ferrier N Metabolic syndrome biomarkers predict lung function impairment: a nested case–control study Am J Respir Crit Care Med 2012 185 4 392 399 Epub 2011/11/19 10.1164/rccm.201109-1672OC 22095549 PMC3297095

[B6] Nolan A Naveed B Comfort AL Ferrier N Hall CB Kwon S Inflammatory biomarkers predict airflow obstruction after exposure to World Trade Center dust Chest 2011 142 2 412 8 Epub 2011/10/15 21998260 10.1378/chest.11-1202 PMC3425337

[B7] Hunninghake GM Cho MH Tesfaigzi Y Soto-Quiros ME Avila L Lasky-Su J MMP12, lung function, and COPD in high-risk populations N Engl J Hum Serv 2009 361 27 2599 2608 Epub 2009/12/19 10.1056/NEJMoa0904006 PMC2904064 20018959

[B8] Rosas IO Richards TJ Konishi K Zhang Y Gibson K Lokshin AE MMP1 and MMP7 as potential peripheral blood biomarkers in idiopathic pulmonary fibrosis PLoS Med 2008 5 4 e93 Epub 2008/05/02 10.1371/journal.pmed.0050093 18447576 PMC2346504

[B9] Jones CB Sane DC Herrington DM Matrix metalloproteinases: a review of their structure and role in acute coronary syndrome Cardiovasc Res 2003 59 4 812 823 Epub 2003/10/14 10.1016/S0008-6363(03)00516-9 14553821

[B10] Death AK Nakhla S McGrath KC Martell S Yue DK Jessup W Nitroglycerin upregulates matrix metalloproteinase expression by human macrophages J Am Coll Cardiol 2002 39 12 1943 1950 Epub 2002/06/27 10.1016/S0735-1097(02)01907-1 12084592

[B11] Imai K Dalal SS Chen ES Downey R Schulman LL Ginsburg M Human collagenase (matrix metalloproteinase-1) expression in the lungs of patients with emphysema Am J Respir Crit Care Med 2001 163 3 Pt 1 786 791 Epub 2001/03/20 11254539 10.1164/ajrccm.163.3.2001073

[B12] Geraghty P Dabo AJ D’Armiento J TLR4 protein contributes to cigarette smoke-induced matrix metalloproteinase-1 (MMP-1) expression in chronic obstructive pulmonary disease J Biol Chem 2011 286 34 30211 30218 Epub 2011/07/07 10.1074/jbc.M111.238824 21730072 PMC3191060

[B13] Vandenbroucke RE Dejonckheere E Libert C A therapeutic role for matrix metalloproteinase inhibitors in lung diseases? Eur Respir J 38 5 1200 1214 Epub 2011/06/11 21659416 10.1183/09031936.00027411

[B14] Martin MD Matrisian LM The other side of MMPs: protective roles in tumor progression Cancer Metastasis Rev 2007 26 3–4 717 724 Epub 2007/08/25 17717634 10.1007/s10555-007-9089-4

[B15] Elkington PT Friedland JS Matrix metalloproteinases in destructive pulmonary pathology Thorax 2006 61 3 259 266 Epub 2005/10/18 10.1136/thx.2005.051979 16227332 PMC2080735

[B16] Joos L He JQ Shepherdson MB Connett JE Anthonisen NR Pare PD The role of matrix metalloproteinase polymorphisms in the rate of decline in lung function Hum Mol Genet 2002 11 5 569 576 Epub 2002/03/05 10.1093/hmg/11.5.569 11875051

[B17] Gosselink JV Hayashi S Elliott WM Xing L Chan B Yang L Differential expression of tissue repair genes in the pathogenesis of chronic obstructive pulmonary disease Am J Respir Crit Care Med 2010 181 12 1329 1335 Epub 2010/01/16 10.1164/rccm.200812-1902OC 20075389 PMC2894408

[B18] Maclay JD McAllister DA Rabinovich R Haq I Maxwell S Hartland S Systemic elastin degradation in chronic obstructive pulmonary disease Thorax 2012 67 7 606 612 Epub 2012/03/01 10.1136/thoraxjnl-2011-200949 22374923

[B19] Weiden MD Ferrier N Nolan A Rom WN Comfort A Gustave J Obstructive airways disease with air trapping among firefighters exposed to World Trade Center dust Chest 2010 137 3 566 574 Epub 2009/10/13 10.1378/chest.09-1580 19820077 PMC2832867

[B20] Weiden MD Naveed B Kwon S Cho SJ Comfort AL Prezant DJ Cardiovascular biomarkers predict susceptibility to lung injury in World Trade Center dust-exposed firefighters Eur Respir J 2013 41 5 1023 30 Epub 2012/08/21 10.1183/09031936.00077012 22903969 PMC3642231

[B21] Rundle AG Vineis P Ahsan H Design options for molecular epidemiology research within cohort studies Cancer Epidemiol Biomarkers Prev 2005 14 8 1899 1907 Epub 2005/08/17 10.1158/1055-9965.EPI-04-0860 16103435

[B22] Shaker SB von Wachenfeldt KA Larsson S Mile I Persdotter S Dahlback M Identification of patients with chronic obstructive pulmonary disease (COPD) by measurement of plasma biomarkers Clin Respir J 2008 2 1 17 25 Epub 2008/01/01 10.1111/j.1752-699X.2007.00032.x 20298300

[B23] Ziora D Dworniczak S Kozielski J Induced sputum metalloproteinases and their inhibitors in relation to exhaled nitrogen oxide and sputum nitric oxides and other inflammatory cytokines in patients with chronic obstructive pulmonary disease J Physiol Pharmacol 2008 59 Suppl 6 809 817 Epub 2009/02/28 19218708

[B24] Engstrom G Lindberg C Gerhardssonde Verdier M Nihlen U Anderson M Svartengren M Blood biomarkers and measures of pulmonary function--a study from the Swedish twin registry Respir Med 2012 106 9 1250 1257 Epub 2012/06/13 10.1016/j.rmed.2012.05.004 22687639

[B25] Ambalavanan N Nicola T Li P Bulger A Murphy-Ullrich J Oparil S Role of matrix metalloproteinase-2 in newborn mouse lungs under hypoxic conditions Pediatr Res 2008 63 1 26 32 Epub 2007/11/29 10.1203/PDR.0b013e31815b690d 18043506 PMC2517580

[B26] Tchougounova E Lundequist A Fajardo I Winberg JO Abrink M Pejler G A key role for mast cell chymase in the activation of pro-matrix metalloprotease-9 and pro-matrix metalloprotease-2 J Biol Chem 2005 280 10 9291 9296 Epub 2004/12/24 15615702 10.1074/jbc.M410396200

[B27] Kheradmand F Kiss A Xu J Lee SH Kolattukudy PE Corry DB A protease-activated pathway underlying Th cell type 2 activation and allergic lung disease J Immunol 2002 169 10 5904 5911 Epub 2002/11/08 12421974 10.4049/jimmunol.169.10.5904

[B28] Caplan-Shaw CE Yee H Rogers L Abraham JL Parsia SS Naidich DP Lung pathologic findings in a local residential and working community exposed to World Trade Center dust, gas, and fumes J Occup Environ Med 2011 53 9 981 991 Epub 2011/08/24 10.1097/JOM.0b013e31822fff60 21860325

[B29] Corry DB Rishi K Kanellis J Kiss A Song Lz LZ Xu J Decreased allergic lung inflammatory cell egression and increased susceptibility to asphyxiation in MMP2-deficiency Nat Immunol 2002 3 4 347 353 Epub 2002/03/12 10.1038/ni773 11887181 PMC2814346

[B30] Gonzalez-Lopez A Astudillo A Garcia-Prieto E Fernandez-Garcia MS Lopez-Vazquez A Batalla-Solis E Inflammation and matrix remodeling during repair of ventilator-induced lung injury Am J Physiol Lung Cell Mol Physiol 2011 301 4 L500 L509 Epub 2011/07/12 10.1152/ajplung.00010.2011 21743031

[B31] D’Armiento J Dalal SS Okada Y Berg RA Chada K Collagenase expression in the lungs of transgenic mice causes pulmonary emphysema Cell 1992 71 6 955 961 Epub 1992/12/11 10.1016/0092-8674(92)90391-O 1458541

